# Mitochondrial dynamics in skin health and disease: energy, aging, and therapeutic perspectives

**DOI:** 10.1093/burnst/tkag008

**Published:** 2026-01-13

**Authors:** Rong Zhang, Tianhao Li, Fengzhou Du, Jiuzuo Huang, Nanze Yu, Xiao Long

**Affiliations:** Department of Plastic and Aesthetic Surgery, Peking Union Medical College Hospital, Chinese Academy of Medical Sciences and Peking Union Medical College, No. 41, Dashucang Hutong, Xicheng District, Beijing 100032, China; Department of Plastic and Aesthetic Surgery, Peking Union Medical College Hospital, Chinese Academy of Medical Sciences and Peking Union Medical College, No. 41, Dashucang Hutong, Xicheng District, Beijing 100032, China; Department of Plastic and Aesthetic Surgery, Peking Union Medical College Hospital, Chinese Academy of Medical Sciences and Peking Union Medical College, No. 41, Dashucang Hutong, Xicheng District, Beijing 100032, China; Center for Regenerative Medicine and Plastic Surgery Research, Peking Union Medical College Hospital, No. 41, Dashucang Hutong, Xicheng District, Beijing 100032, China; Department of Plastic and Aesthetic Surgery, Peking Union Medical College Hospital, Chinese Academy of Medical Sciences and Peking Union Medical College, No. 41, Dashucang Hutong, Xicheng District, Beijing 100032, China; Center for Regenerative Medicine and Plastic Surgery Research, Peking Union Medical College Hospital, No. 41, Dashucang Hutong, Xicheng District, Beijing 100032, China; Department of Plastic and Aesthetic Surgery, Peking Union Medical College Hospital, Chinese Academy of Medical Sciences and Peking Union Medical College, No. 41, Dashucang Hutong, Xicheng District, Beijing 100032, China; Center for Regenerative Medicine and Plastic Surgery Research, Peking Union Medical College Hospital, No. 41, Dashucang Hutong, Xicheng District, Beijing 100032, China; Department of Plastic and Aesthetic Surgery, Peking Union Medical College Hospital, Chinese Academy of Medical Sciences and Peking Union Medical College, No. 41, Dashucang Hutong, Xicheng District, Beijing 100032, China; Center for Regenerative Medicine and Plastic Surgery Research, Peking Union Medical College Hospital, No. 41, Dashucang Hutong, Xicheng District, Beijing 100032, China

**Keywords:** Mitochondrial, Dynamics, Skin, Health, Diseases, Energy

## Abstract

As cellular energy metabolic hubs, mitochondria undergo dynamic fusion–fission cycles and autophagy that enable rapid adaptation to cellular energy demands and stress conditions. In addition to their role in energy metabolism, mitochondria are integral to cellular homeostasis and regulate cell cycle progression, differentiation, and apoptosis pathways. In recent years, the importance of mitochondrial function in skin health and disease has garnered increasing attention. Mitochondrial dysfunction has been implicated in a spectrum of skin disorders, including skin aging, psoriasis, vitiligo, keloids, scleroderma, and skin cancer. The pathogenesis of these conditions is closely linked to mitochondrial deoxyribonucleic acid (mtDNA) damage, excessive reactive oxygen species (ROS) production, and alterations in mitochondrial metabolic pathways. In terms of therapeutic strategies, this review summarizes a range of mitochondrion-targeted interventions. These treatments include the activation of the PGC-1α pathway to increase mitochondrial adenosine triphosphate synthesis, the use of antioxidants to mitigate mitochondrial ROS production, and the application of bioactive compounds and drugs to protect mitochondria or promote mtDNA repair. These approaches not only contribute to improved skin health but also provide novel insights for the treatment of skin diseases. Additionally, mitochondrial transplantation technology has shown considerable promise in skin regeneration and wound healing and is emerging as a new frontier for skin tissue repair.

## Highlights

- Crucial role of mitochondria in skin health: Mitochondria are the center of energy metabolism in skin cells. They are vital for preserving the integrity of the skin barrier and are involved in the regulation of the cell cycle and differentiation processes. Mitochondrial dysfunction can precipitate a range of skin conditions, including premature aging, psoriasis, vitiligo, and impaired wound healing.
- Dermatological treatments targeting mitochondria: Multiple strategies are being explored to harness mitochondrial function to manage skin diseases. These treatments include increasing mitochondrial adenosine triphosphate synthesis to increase metabolic activity and employing antioxidants to curb reactive oxygen species (ROS) production or to neutralize existing ROS, thereby safeguarding mitochondrial integrity.
- Future research on mitochondrial targeted therapy will focus on mitochondrial dynamics, mitochondrial autophagy, and the interaction between mitochondria and cell death pathways. The challenges to be addressed include how to precisely target mitochondria, how to evaluate the status of mitochondrial function, and how to balance treatment efficiency.

## Background

As the largest organ of the human body, the skin requires considerable amounts of energy to support its wide range of physiological functions, with mitochondrial activity forming the foundation of these processes. In addition to their fundamental role in energy production, mitochondria actively contribute to the regulation of cellular fate, the regulation of biochemical signaling pathways, and the maintenance of internal homeostasis. Efficient mitochondrial function is essential for preserving skin integrity and mitigating the development of various pathological conditions.

### Structure and biological functions of the skin

The skin is the largest organ of the human body and provides protection from the external environment while performing various functions, such as immunity, temperature regulation, sensation, absorption, and excretion [1]. The outermost layer, the epidermis, consists mainly of keratinocytes that form a protective barrier to prevent water loss and the intrusion of external substances. The dermis lies beneath the epidermis and contains collagen, elastic, and reticular fibers, along with other components that are essential for skin elasticity, strength, and sensation. Subcutaneous tissue, which is primarily composed of adipose tissue, contributes to energy storage and insulation and contains blood vessels, lymphatics, nerves, and skin appendages [2]. Cells across all skin layers continuously proliferate, differentiate, and undergo programmed death to ensure the protective function, elasticity, softness, and overall health of the skin. As a biologically active system, the skin plays a vital role in overall health and directly influences an individual’s quality of life [3].

### Definition and general function of mitochondria

Mitochondria are essential cellular organelles responsible for energy conversion. They produce adenosine triphosphate (ATP) through the tricarboxylic acid cycle and oxidative phosphorylation, supporting various cellular activities [4]. These organelles are dynamic and can adapt to varying metabolic demands [5]. Recent research has shown that mitochondria also contribute to cell fate determination and aging. In addition to energy metabolism, they are involved in cell signaling, cell cycle regulation, and apoptosis. By integrating processes such as biogenesis, fission, fusion, proteolysis, and autophagy, mitochondria possess complex quality control mechanisms that regulate their morphology and function. These mechanisms help preserve mitochondrial deoxyribonucleic acid (DNA) integrity and electrochemical connectivity, ensuring mitochondrial homeostasis, which is vital for normal cell function and health. Thus, these regulatory mechanisms are integral to cellular physiology [6].

### The link between skin health and mitochondria

**Figure 1:**
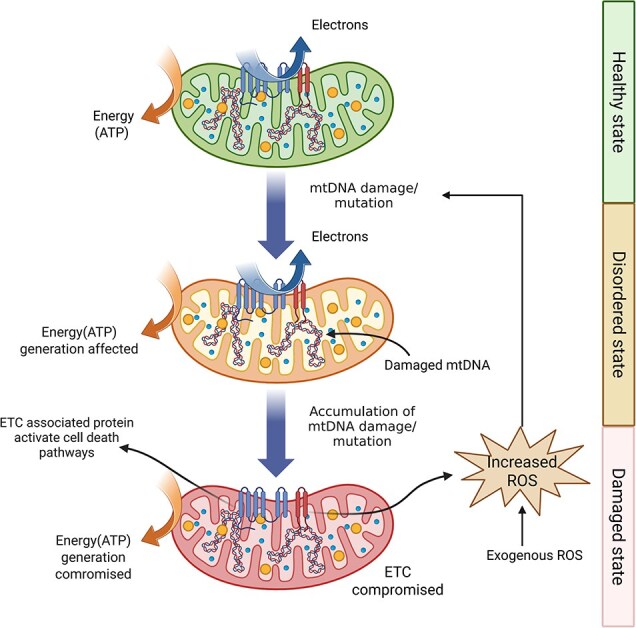
The electron chain releases free radicals, causes mtDNA damage, and leads to a vicious cycle of ROS-mediated mitochondrial loss. *ATP* adenosine triphosphate, *ROS* reactive oxygen species, *ETC* electron transport chain. Created at https://BioRender.com

Mitochondria serve as central regulators of the generation of reactive oxygen species (ROS) and the control of apoptosis pathways. Disruptions in the equilibrium between ROS generation and cellular antioxidant mechanisms may trigger oxidative injury to cellular components such as DNA, proteins, and lipid membranes within cutaneous tissues (Figure 1). These pathological alterations are clinically linked to photodermatoses, including polymorphic light eruption and solar urticaria. Emerging research highlights that maintaining proper mitochondrial dynamics and functional integrity represents a vital therapeutic strategy for managing these dermatological conditions [7].

### The purpose of this review

This review aims to explore the role of mitochondria in maintaining skin health, including their involvement in cellular energy metabolism, skin barrier function, cell cycle regulation, and differentiation, and the relationship between mitochondrial dysfunction and various skin diseases, with the goal of identifying mitochondria as potential therapeutic targets in dermatological disorders.

## Review

### Mitochondria and skin health

As central regulators of skin biology, mitochondria fulfill dual functions by acting as bioenergetic hubs that drive biosynthetic processes in keratinocytes and dermal fibroblasts while also orchestrating cellular differentiation through redox signaling and ion homeostasis. Furthermore, these organelles mediate immunometabolic crosstalk, which plays a crucial role in sustaining and fine-tuning the immune defense mechanisms of the skin.

#### Role of mitochondria in energy metabolism in skin cells

The epidermis is a metabolically dynamic tissue, and its regenerative potential fundamentally relies on the proliferation of progenitor cells. Mitochondrial oxidative phosphorylation constitutes the principal energy supply for these rapidly dividing cell populations.

(1) Skin cells, especially keratinocytes and hair follicle dermal papilla cells, are highly energy dependent. Their renewal relies on the rapid proliferation of precursor cells, making them especially sensitive to the accumulation of DNA mutations and mitochondrial dysfunction. Sanjit K. Dhar *et al.* showed that treating primary human keratinocytes with MitoParaquat significantly increases mitochondrial superoxide levels, whereas basal respiration, maximal respiration, reserve capacity, and ATP-related energy production are significantly reduced. These mitochondrial impairments ultimately compromise keratinocyte function, leading to weakened skin barrier integrity and delayed wound healing [8].
(2) Fibroblasts in the dermis are responsible for synthesizing and maintaining collagen in the skin, a process that is also highly energy intensive. Qifei Wang *et al.* reported that under hypoxic conditions, fibroblasts in keloid tissue exhibit upregulated HIF-1ɑ protein levels and increased phosphorylation of proteins in the PI3K/AKT signaling pathway. As a result, mitochondrial function is impaired, and autophagy increases [9]. When mitochondrial function is compromised, fibroblasts fail to generate sufficient energy, leading to reduced collagen production, decreased skin elasticity, and the formation of wrinkles [10].

#### Role of mitochondria in skin cell proliferation and maturation

Mitochondria are involved not only in energy production but also in regulating the cell cycle and cellular differentiation. Oxidative stress in skin cells can affect the cell cycle by altering the activity of cytokines through transcription factors such as p16INK4A and p53 [11]. The mitochondrial transcription factor TFAM plays an essential role in mitochondrial deoxyribonucleic acid (mtDNA) replication. Studies have shown that cellular models with TFAM deficiency display decreased efficiency of oxidative phosphorylation and reduced ROS generation, highlighting the critical involvement of both mitochondrial energy metabolism and redox signaling in keratinocyte differentiation. In vivo models further support this finding, as conditional TFAM knockout mice exhibit increased perinatal mortality linked to defective skin barrier development. Additionally, the differentiation potential of epidermal cells isolated from these genetically altered mice is significantly impaired under in vitro culture conditions [12].

Mitochondrial ROS generation plays a pivotal regulatory role in the differentiation of various cell lines. Elevated ROS levels promote the differentiation of mouse embryonic stem cells (ESCs), induced pluripotent stem cells (iPSCs), and tissue-specific pluripotent epithelial stem cells. In contrast, reduced ROS levels are associated with increased mitotic activity. In skin cells, low concentrations of hydrogen peroxide (H_2_O_2_) can activate growth factor receptors on keratinocytes, thereby promoting keratinization.

Moreover, mitochondrial respiration relies on the presence of calcium ions (Ca^2+^). The Ca^2+^ gradient observed in human skin suggests a close relationship between mitochondrial respiration and keratinocyte differentiation [13]. In vitro studies have shown that increasing extracellular Ca^2+^ concentrations enhance keratinocyte differentiation. Conversely, the inhibition of mitochondrial Ca^2+^ uptake impairs this process, indicating that Ca^2+^ plays a crucial role in regulating mitochondrial function and, consequently, skin cell differentiation [14].

#### Immunometabolic interactions between mitochondria and immune cells in the skin

Mitochondria play foundational roles not only in cellular energy metabolism but also in the regulation of immune cell function. In the skin, immune components such as dendritic cells, Langerhans cells, and T cells form an integral immunological network, and their activation, differentiation, and effector functions are strongly influenced by the mitochondrial metabolic state. Dendritic cells, including Langerhans cells, rely on mitochondrial oxidative phosphorylation and a proper redox balance to support antigen presentation and immune tolerance. The disruption of mitochondrial homeostasis and impaired autophagy can attenuate dendritic cell function, thereby compromising immune surveillance and tolerance induction in the skin [15, 16]. T cells are equally metabolically demanding: their activation and differentiation require a dynamic metabolic switch between oxidative phosphorylation and glycolysis. Dysregulation of mitochondrial metabolism can skew T-cell subsets toward proinflammatory (Th17) cells over regulatory T cells (Tregs), which has been implicated in inflammatory skin conditions such as psoriasis and allergic dermatitis. Single-cell immunometabolic analyses of psoriatic skin further revealed that increased activity of metabolic pathway [17, 18], fatty acid degradation and ascorbate metabolism are associated with Treg function, suggesting a protective metabolic signature, whereas proinflammatory T-cell subsets exhibit distinct metabolic reprogramming [19].

#### Role of mitochondria in skin pigmentation

Mitochondria play a vital role in supporting melanocyte function by supplying the ATP necessary for various energy-dependent processes, including melanin synthesis.

##### Melanocyte function and mitochondria

Melanocytes, located in the basal layer of the epidermis, are the primary cells responsible for pigment production in skin. These cells contain melanosomes, specialized organelles that synthesize melanin, the major pigment in the skin, hair, and eyes [20]. Given the high metabolic activity associated with melanosome biogenesis and melanin production, melanocytes have substantial energy demands.

The mitochondrial electron transport chain is not only responsible for ATP generation but also produces ROS, such as superoxide and H_2_O_2_, which are known to be involved in the regulation of melanin synthesis. These ROS can act as signaling molecules that influence the activity of enzymes involved in melanogenesis, such as tyrosinase.

Nicotinamide nucleotide transhydrogenase (NNT), a mitochondrial enzyme, plays a key role in maintaining the cellular redox state by catalyzing the transhydrogenation reaction between NADPH and NAD+. Alterations in NNT activity can affect the redox state of melanocytes, influencing the degradation of tyrosinase and thereby regulating melanosome maturation. This process, in turn, affects the production of eumelanin and the overall degree of skin pigmentation [21]. Mutations or dysfunction in mtDNA can have profound effects on melanocyte function and melanin synthesis. Such alterations can disrupt the electron transport chain, leading to decreased ATP production and increased ROS generation, which can disrupt melanogenesis and alter pigmentation [22]. For instance, mtDNA mutations have been implicated in disorders such as oculocutaneous albinism, which is characterized by reduced pigmentation of the skin, hair, and eyes.

##### Mitochondrial regulation of melanin synthesis

Mitochondria regulate melanin synthesis through a multifaceted set of mechanisms. ROS, which are produced as byproducts of the mitochondrial electron transport chain, play a key role in activating tyrosinase, the enzyme responsible for catalyzing the initial step of melanin synthesis. The activity of tyrosinase is tightly regulated by the intracellular redox balance, which is directly influenced by the mitochondrial functional status. A well-maintained redox environment is essential for sustaining the ROS–ERK (extracellular signal-regulated kinase) signaling pathway, which is a critical regulator of melanogenesis [23].

Furthermore, mitochondria may indirectly control melanin synthesis by modulating the differentiation and maturation of melanocytes. These cells undergo complex developmental processes involving dynamic changes in gene expression and cellular morphology, ultimately resulting in the production of mature melanosomes filled with melanin. Mitochondrial dysfunction has been shown to disrupt these processes, potentially leading to abnormal melanin synthesis and altered pigmentation patterns.

Interestingly, polydeoxynucleotide (PDRN), a compound derived from marine sources, has been shown to regulate melanin synthesis in melanocytes. Specifically, PDRN significantly reduces the melanin content in B16-F10 melanocytes by directly inhibiting the activity of both mushroom tyrosinase and cellular tyrosinase. Additionally, PDRN downregulates the mRNA and protein expression of microphthalmia-associated transcription factor (MITF), a key regulator of melanocyte differentiation and melanogenesis. Through MITF suppression, PDRN further reduces the expression of tyrosinase-related protein 1 (TRP1), dopachrome tautomerase (TRP2), and tyrosinase itself, all of which are essential enzymes in the melanin biosynthesis pathway [24].

### Mitochondria and skin diseases

Mitochondrial dysfunction represents a central pathological mechanism underlying a range of dermatological disorders, such as age-associated degeneration, neoplastic development, inflammatory reactions, fibrotic remodeling, and dysregulated melanin synthesis. These pathophysiological effects are driven primarily by impaired ATP production, excessive ROS accumulation, mitochondrial genomic instability, and consequent immune activation. The molecular pathways associated with these disease processes provide promising targets for the development of innovative therapeutic strategies.

#### Mitochondrial dysfunction and skin pathology

Mitochondrial structural and functional abnormalities, such as a reduced membrane potential, disrupted the electron transport chain activity, and the accumulation of mtDNA mutations, have been shown to impair cellular energy metabolism, increase ROS production, and undermine skin homeostasis. Notably, many of these mitochondrial alterations are reversible following the restoration of mitochondrial function, highlighting their pivotal roles in cutaneous health [25]. Skin aging exemplifies the downstream pathology. Structurally, the skin thins, dries, develops uneven pigmentation, and wrinkles. Mechanistically, these features are associated with insufficient ATP production, increased ROS generation, and mitochondrial respiration defects. Aging skin exhibits a decreased mtDNA copy number and increased mutational burden, which together disrupt respiratory chain efficiency, impair mitochondrial biogenesis, and compromise protein synthesis [26]. Additionally, elevated ROS levels activate stress-responsive transcription factors such as NF-κB and MAPK, which in turn upregulate matrix metalloproteinases (MMPs), leading to increased collagen and elastin degradation in the extracellular matrix (ECM) and thus visible signs of skin aging [27].

In addition to aging, mitochondrial dysfunction underlies a range of skin pathologies, including inflammation, fibrosis, impaired wound healing, pigmentary disorders, and skin cancers. For instance, oxidized mtDNA fragments can escape into the cytosol and act as DAMPs, activating innate immune signaling pathways such as the cGAS–STING and TLR9 pathways, which are involved in chronic inflammatory skin diseases such as psoriasis and atopic dermatitis (AD) [28]. Dysfunctional mitochondrial metabolism also affects melanocyte viability, fibroblast activation, immune cell polarization, and tumor cell survival, highlighting the multifaceted role of these organelles in skin disease development.

#### Degeneration of mitochondrial function during aging

A gradual decrease in mitochondrial efficiency and function leads to reduced energy production and the accumulation of free radicals, which in turn affect the health and functionality of cells and tissues. The accumulation of oxidative DNA damage and dysfunctional mitochondria disrupts cellular homeostasis and contributes to increased apoptosis. Studies have shown that alterations in DNA repair pathways and the induction of mitochondrial dysfunction can result in genetic disorders associated with premature aging [29]. In naturally aged skin, CRAT (carnitine acetyltransferase) expression decreases significantly. Experimental knockdown of CRAT in human dermal fibroblasts (HDFs) leads to mitochondrial damage and dysfunction, reducing cell proliferation and increasing the number of senescent cells [30]. This deficiency alters mitochondrial morphology and metabolism, causing mtDNA damage. Impaired CRAT expression hinders fatty acid transport and metabolism, reducing β-oxidation and ATP production [31]. Consequently, mtDNA is released into the cytoplasm, where it activates the cGAS–STING and NF-κB signaling pathways, ultimately inducing senescence-associated secretory phenotypes (SASPs).

The release of mitochondrial DNA into the cytoplasm may also function as a damage-associated molecular pattern (DAMP), triggering inflammatory signaling pathways and the expression of SASP-related genes. These changes not only affect the cellular energy supply but also increase intracellular oxidative stress, thereby accelerating the skin aging process [32].

Because mtDNA lacks histone protection and has a limited DNA repair capacity [33], it is highly susceptible to damage-induced mutation [34]. The increased mutation rate of mtDNA further compromises mitochondrial function and accelerates the skin aging process [35, 36]. These mutations can impair mitochondrial protein synthesis, affecting the integrity and efficiency of the respiratory chain and leading to dysfunction in skin cells. Fibroblasts derived from older adults exhibit a relatively high frequency of mtDNA point mutations, suggesting a strong link between mtDNA damage and skin aging [37].

**Figure 2:**
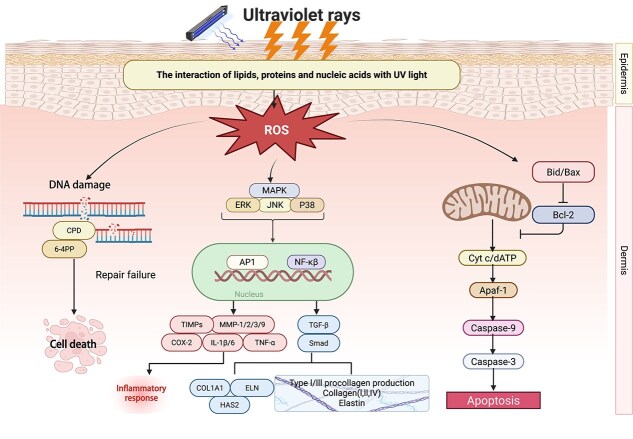
The signaling pathway of ROS in active UV-induced photodamage of the skin. *ROS* reactive oxygen species, *UV* Ultraviolet, *NF-κB* nuclear factor kappa-light-chain-enhancer of activated B cells, *TNF* tumor necrosis factor, *TGF-β* transforming growth factor-β, *IL* interleukin. Created at https://BioRender.com

Mitochondria are the primary sites of both energy metabolism and ROS production, making mtDNA particularly sensitive to oxidative stress [38]. Excessive ROS can damage mitochondrial proteins, lipids, and DNA, further exacerbating mitochondrial dysfunction and creating a vicious cycle. ROS also activate the mitogen-activated protein kinase (MAPK) signaling pathway, leading to the upregulation of MMPs, which play a key role in skin aging through the degradation of collagen and elastin in the ECM [39]. In addition, ROS can directly damage ECM components in skin cells, causing the skin to lose elasticity and structural integrity, which are characteristics of skin aging (Fig. 2) [40].

##### Targeting mitochondrial deoxyribonucleic acid mutations: mechanisms and rescue strategies

Studies have shown that the accumulation of mtDNA mutations not only impairs oxidative phosphorylation and compromises the integrity of the respiratory chain but also triggers stress response pathways such as the mitochondrial unfolded protein response (UPR^mt), thereby exacerbating inflammation and subsequent mitochondrial dysfunction. For instance, defective replication or proofreading of mtDNA caused by mutations in the POLG gene, which encodes mitochondrial DNA polymerase, has been linked to premature skin aging and various mitochondrial syndromes [41].

Several mitochondrial rescue strategies have been proposed to counteract these pathological processes. Supplementation with NAD+ precursors, such as nicotinamide riboside (NR) or nicotinamide mononucleotide (NMN), has been shown to increase mitochondrial biogenesis and promote DNA repair in aging skin cells [42]. In addition, pyrroloquinoline quinone (PQQ), a redox cofactor, has been reported to stimulate mitochondrial biosynthesis and protect mtDNA from oxidative damage. More recently, mitochondrion-targeted genome editing technologies, such as DddA-derived cytosine base editors (DdCBEs), have been shown to selectively correct pathogenic mtDNA mutations, although they are currently being investigated at a preclinical stage.

##### Application of mitochondrial protection strategies in sunscreen and anti-aging products

Given the central role of mitochondria in skin photoaging, the development of sunscreen and anti-aging products that target mitochondrial protection is of increasing importance. Guo K *et al.* found that [43] cryptotanshinone (CTS) promotes mitochondrial biosynthesis by activating AMPK/SIRT1/PGC-1α signaling, thereby improving mitochondrial function, reducing cell death, and effectively delaying UV-induced senescence in skin cells. These findings provide both theoretical and experimental support for the use of CTS as a potential mitochondrion-targeting agent in sunscreen and anti-aging formulations. Similarly, fucoidan polysaccharides extracted from *Laminaria japonica* have been shown to increase mitochondrial biogenesis in HaCaT and HFF-1 cells via the SIRT1/PGC-1α signaling axis, inhibit ROS production, and exert antiphotoaging effects [44]. Eltania F. *et al.* demonstrated that a tranexamic acid (TA) cream inhibits UVB-induced oxidative stress and inflammation, suppresses ROS generation, and downregulates inflammatory mediators such as IL-6 and TNF-α. Furthermore, TA upregulated the expression of mitochondrial biogenesis-related genes and enhanced mitochondrial function and cellular energy metabolism. In addition, several natural antioxidants, including vitamin E, vitamin C, and flavonoids, have been reported to mitigate UV-induced mitochondrial damage and oxidative stress in skin cells [45]. Other compounds, such as resveratrol and green tea extract, are also believed to exert photoprotective effects by activating mitochondrial antioxidant pathways and improving mitochondrial resilience under oxidative stress [46].

#### Tumorigenic skin disorders

Mitochondria play crucial roles in the development of skin cancer through mtDNA mutations, dysregulation, and metabolic reprogramming. Furthermore, they represent promising targets for innovative therapies designed to selectively induce tumor cell death.

##### Skin cancer

Mitochondria contribute centrally to skin carcinogenesis through multiple interrelated mechanisms. Somatic mutations in mtDNA, which are common in various skin cancers, alter mitochondrial function and favor a shift toward aerobic glycolysis, which is commonly known as the Warburg effect. A recent study demonstrated that mtDNA mutations in melanoma cells drive increased glycolysis, which not only supports tumor metabolism but also may increase responsiveness to immune checkpoint blockade therapy [47]. Moreover, broader evidence indicates that the accumulation of mtDNA mutations in tumors disrupts oxidative phosphorylation and exacerbates oxidative stress, reinforcing a feedback loop that supports malignant progression [48].

Beyond genomic alterations, mitochondrial dynamics, namely, the balance between fusion and fission, are dysregulated in skin cancers. Detailed studies of melanoma cells have revealed that interfering with fission via the inhibition of Drp1 or treatment with Mdivi-1 not only promotes mitochondrial fusion but also influences cell viability and migratory behavior, affecting tumor progression [49]. Broad reviews in oncology further suggest that altered mitochondrial dynamics increase the vulnerability of cancer cells and represent promising therapeutic targets [50].

Metabolic reprogramming in skin cancers also stems from oncogene-driven mitochondrial adjustments. In melanomas harboring the BRAFV600E mutation, constitutive MAPK pathway activation leads to the suppression of the mitochondrial oxidative phosphorylation regulators PGC-1ɑ and MITF and the promotion of glycolysis through the stabilization of HIF-1ɑ, further reinforcing the glycolytic phenotype and supporting tumor survival and progression [51].

##### Mitochondrion-targeted therapies for skin cancer

Therapeutic strategies that directly target mitochondrial function are emerging as innovative approaches for skin cancer treatment. One promising avenue involves exploiting lysosomal zinc (Zn^2+^) dynamics: in metastatic melanoma cells, lysosomal channels such as TRPML1 are markedly upregulated, and pharmacological activation of these channels triggers rapid, mitochondria-mediated, necrotic cell death through excessive Zn^2+^ release while sparing normal cells. This Zn^2+^-dependent cytotoxicity results in mitochondrial swelling, ATP depletion, and effective tumor suppression in experimental melanoma models [52].

Another mitochondria-centered strategy utilizes the immune response modulator imiquimod, which induces ROS production in skin cancer cells, resulting in a loss of the mitochondrial membrane potential, increased mitochondrial fragmentation, and increased mitophagy, thereby disrupting mitochondrial integrity in tumor cells. The mitochondrial stress and structural remodeling that occur after imiquimod treatment may contribute to its antitumor effects [53].

In addition to these targeted interventions, melatonin and its metabolites have been shown to exert protective and regulatory effects on mitochondria in tumors. In melanoma cell models, melatonin enhances mitochondrial quality control by promoting the expression of fusion-regulating proteins, mitigating oxidative damage, and supporting mitochondrial energy homeostasis. These actions may render tumor cells more susceptible to stress and reduce their proliferative potential [54, 55].

#### Inflammatory skin diseases

Mitochondrial dysfunction activates the innate immune response by releasing mitochondrial DNA as a DAMP, thereby exacerbating chronic skin inflammation conditions such as psoriasis and AD. Additionally, it induces oxidative stress, metabolic reprogramming, and disruption of the skin barrier, opening avenues for targeted antioxidant and immunometabolic therapies.

##### Psoriasis

Psoriasis is a chronic inflammatory dermatological disease characterized by aberrant keratinocyte proliferation and sustained immune system activation. Emerging evidence reveals that mitochondrial dysfunction critically contributes to its pathogenesis through multiple, interlinked mechanisms. Notably, studies have reported a decrease in the mitochondrial DNA copy number (mtDNA-CN) in peripheral blood from psoriasis patients, with lower levels significantly correlated with the disease duration and severity and suggesting their potential use as a non-invasive biomarker for disease progression [56].

Mechanistically, mtDNA released into the cytoplasm functions as a DAMP, activating innate immune pathways such as the cGAS–STING pathway and AIM2 inflammasome and thereby initiating robust inflammatory signaling and type I interferon responses in keratinocytes and immune cells, which are key drivers of psoriatic inflammation [57, 58]. Dysregulated mitochondrial metabolism further fuels ROS production and activates NF-κB, reinforcing inflammatory networks and perturbing epidermal homeostasis.

Immune–metabolic crosstalk also plays a central role. Mitochondrial dysfunction in dendritic cells and keratinocytes can skew T-cell responses toward Th17 differentiation through the increased production of IL-1β, IL-6, and IL-23. In fact, in imiquimod-inducible murine psoriasis models, impaired mitophagy linked to defective AMPK signaling exacerbates keratinocyte proliferation and inflammation, while its restoration mitigates psoriatic pathology, highlighting the importance of mitochondrial quality control in disease control.

##### Atopic dermatitis

AD is a chronic inflammatory skin disorder in which mitochondrial dysfunction has emerged as a key pathogenic factor. Notably, even in the nonlesional (apparently normal) epidermis of AD patients, keratinocytes display increased mitochondrial activity, including increased oxidative phosphorylation, fatty acid oxidation, and TCA cycle turnover, leading to increased ROS production and oxidative stress [59]. This hypermetabolic state disrupts keratinocyte function, impairs lamellar body formation, and compromises the epidermal barrier, setting the stage for inflammation.

Concomitant with mitochondrial hyperactivity, proteomic studies have revealed reduced activity of NRF2, a master regulator of antioxidant response pathways, in lesional AD skin, further exacerbating oxidative stress and inflammation [60]. In this context, plasma levels of cell-free mtDNA are elevated in AD patients, which correlate with disease severity and suggest a DAMP-mediated amplification of immune responses [61].

Therapeutically, mitochondrion-targeted antioxidant interventions show promise. Treatment of AD-like human epidermal models with MitoQ effectively normalized mitochondrial function, restored lamellar body structure, and reduced NF-κB activation and cellular damage [62]. Experimental compounds such as imidazole propionate, which suppresses mitochondrial ROS production and inhibits mTORC2 signaling, str also effective at ameliorating AD-like lesions in preclinical models [63].

#### Fibrotic skin disorders

Emerging evidence suggests that mitochondrial dysfunction plays an important role in the pathogenesis of fibrotic skin diseases, such as keloids and scleroderma. Under these conditions, bioenergetic deficits, oxidative imbalances, and dysregulated apoptosis contribute to the persistence of fibroblasts and pathological matrix deposition.

##### Keloids and scleroderma

Although keloid and scleroderma are not primary mitochondrial diseases, accumulating evidence suggests that mitochondrial dysfunction may play an indirect yet significant role in their pathogenesis. These disorders share features of abnormal tissue remodeling and chronic inflammation, with the skin being a major affected organ. Clinically, keloids present as excessive fibrotic outgrowths, whereas scleroderma is characterized by widespread dermal thickening, rigidity, and pigmentary changes [64].

The involvement of mitochondrial dysfunction is supported by findings that implicate disruptions in cellular energy metabolism, oxidative balance, and apoptosis regulation in fibrotic processes. These mitochondrial abnormalities may impair cell cycle control and increase fibroblast survival, thereby contributing to the excessive matrix deposition observed in patients with these diseases [65]. While direct mtDNA mutations have not been identified as causative, therapeutic strategies designed to support mitochondrial function, such as the use of antioxidants or mitochondria-protective agents, are under investigation. Although gene therapy targeting mitochondrial genomes is currently not applicable, small molecules that improve mitochondrial integrity may represent future treatment prospects.

#### Pigmentary skin disorders

Mitochondrial dysfunction is a key factor in the development of both hypopigmentation and hyperpigmentation disorders, highlighting novel therapeutic opportunities for the treatment of pigmentary skin diseases.

##### Leukoderma (vitiligo)

Leukoderma, most notably vitiligo, is characterized by the progressive loss of epidermal melanocytes, leading to the formation of depigmented skin patches. Emerging evidence implicates mitochondrial dysfunction in melanocyte degeneration through multiple mechanisms, including energy failure, oxidative stress, and programmed cell death. In vitro studies of cultured melanocytes from individuals with vitiligo have demonstrated significantly lower ATP production and increased mitochondrial proton leakage, indicating an inefficient electron transport chain and compromised bioenergetics [66]. These energy deficits likely render melanocytes more vulnerable to environmental and immunologic stressors.

Under oxidative conditions, the expression of the TRPM2 channel, a calcium-permeable ion channel that is responsive to oxidative stress, is increased in melanocytes from individuals with vitiligo. This upregulation leads to excessive Ca^2+^ influx into mitochondria, triggering apoptosis through mitochondrial depolarization and the activation of intrinsic cell death pathways [67]. In addition to apoptosis, altered mitochondrial metabolism may also predispose melanocytes to ferroptosis and impaired autophagy; however, detailed mechanistic studies remain limited.

From a therapeutic perspective, compounds such as pioglitazone, a PPARγ agonist, have been shown to preserve mitochondrial integrity and increase the survival of stressed melanocytes in preclinical models. Although tretinoin (calcipotriol) is known primarily for increasing melanogenesis, its antioxidant actions in melanocytes suggest its potential utility in ameliorating mitochondrial damage; further in-depth studies are warranted to confirm this effect [68].

##### Melasma and freckles

Melasma and freckles are common hyperpigmentary disorders linked to mitochondrial dysfunction, particularly via oxidative stress pathways. In patients with melasma, elevated serum levels of markers of oxidative damage, such as malondialdehyde, together with disturbed thiol–disulfide homeostasis, underscore a redox imbalance that correlates with disease severity [69]. This oxidative milieu appears to sensitize melanocytes toward increased melanin synthesis and uneven pigment distribution. Excess ROS generation underlies signaling cascades that activate melanogenic enzymes such as tyrosinase, thereby promoting localized hyperpigmentation.

Mitochondrial quality control mechanisms, particularly selective autophagy, play a vital role in regulating melanosome turnover and pigment homeostasis. The disruption of autophagic flux, whether due to impaired mitophagy or broader lysosomal dysfunction, leads to the accumulation of damaged mitochondria and unrestrained melanogenesis, contributing to the development of hyperpigmented lesions. Notably, impaired autophagy is associated with pigmentary imbalances in both melasma and age-related hyperpigmentation, such as senile lentigines [70].

While specific mitochondrial DNA mutations or polymorphisms have not been strongly implicated in melasma or freckles, metabolic disturbances and impaired mitochondrial turnover are emerging as plausible contributors to melanocyte dysfunction. Restoring autophagy and mitochondrial integrity in melanocytes may therefore represent a novel therapeutic approach for managing hyperpigmentation by re-establishing the redox balance and curbing excessive melanin production.

**Figure 3:**
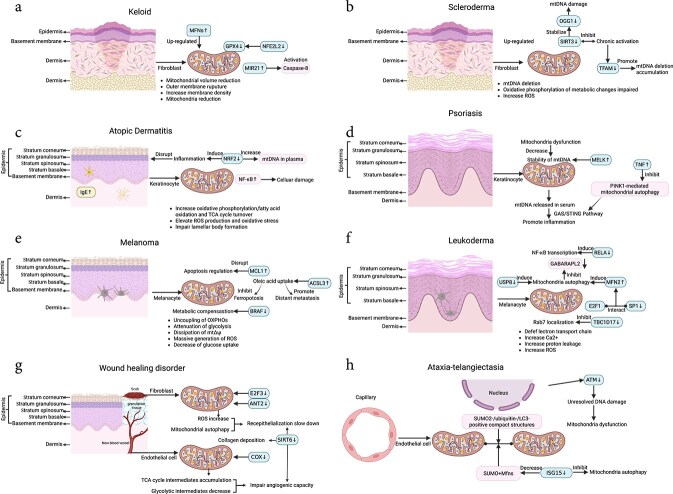
Pathogenesis of skin diseases related to mitochondria. *MFNs* mitofusins, *GPX4* glutathione peroxidase 4, *NFE2L2* nuclear factor erythroid 2-related factor 2, *MIR21* microRNA 21, *mtDNA* mitochondrial DNA, *SIRT3* sirtuin 3, *TFAM* mitochondrial transcription factor A, *ROS* reactive oxygen species, *NF-κB* nuclear factor kappa-light-chain-enhancer of activated B cells, *TCA* tricarboxylic acid, MELK maternal embryonic leucine zipper kinase, *TNF* tumor necrosis factor, *PINK1* PTEN induced putative kinase 1, *GAS* cyclic GMP-AMP synthase, *STING* stimulator of interferon genes, *MCL1* myeloid cell leukemia 1, *ACSL3* Acyl-CoA Synthetase Long-Chain Family Member 3, *E2F1* E2F transcription factor 1, *SP1* specificity protein 1, *Rab7* ras-related protein rab-7, *TBC1D17* TBC1 domain family member 17, *SIRT6* sirtuin 6, COX Cytochrome c oxidase, *SUMO2* small ubiquitin-like modifier 2, *LC3* microtubule-associated protein 1A/1B-light chain 3, *ISG15* interferon-stimulated gene 15, *Mfns* mitofusins, *UV* Ultraviolet, *CPD* cyclobutane pyrimidine dimer, *dATP* deoxyadenosine triphosphate, *Apaf-1* apoptotic protease activating factor 1, *ATP* adenosine triphosphate. Created at https://BioRender.com

To facilitate understanding of mitochondrial damage in different skin diseases, we have now compiled the following table (Table 1) [71–77] and visualized it through images (Fig. 3).

**Table 1:** Mitochondrial damage in different skin diseases

Skin disease	Type of mitochondrial damage	Changes in ROS levels	mtDNA abnormality	Ref.
Melanoma	Dysregulated mitochondrial dynamics (imbalanced fusion/fission), suppressed oxidative phosphorylation, and metabolic reprogramming	mtDNA mutations lead to intensified oxidative stress and elevated ROS levels	Accumulation of somatic mtDNA mutations	[71]
Psoriasis	Dysregulated mitochondrial metabolism and impaired mitophagy	Increased ROS production, which activates NF-κB inflammatory signaling	Reduced mtDNA replication	[72]
Atopic dermatitis	Increased oxidative phosphorylation, fatty acid oxidation, and TCA cycle turnover	Elevated ROS production, leading to oxidative stress	Increased levels of cell-free mtDNA in plasma	[73]
Keloids	Impaired mitophagy, abnormal mitochondrial membrane potential and abnormal mitochondrial morphology	Increased ROS levels promote TGFβ 1-induced phosphorylation of SMAD3 and ERK1/2	No specific mtDNA abnormality was reported	[74]
Scleroderma	Decreased respirasomes and dysregulated mitochondrial fission/fusion	Increased mtROS production and upregulated MnSOD expression	mtDNA damage with reduced expression of the DNA repair enzyme OGG1	[75]
Leukoderma	Reduced ATP production, increased proton leakage, an inefficient electron transport chain, and Ca^2+^-induced mitochondrial depolarization	Upregulation of TRPM2 under oxidative stress, amplifying ROS-mediated stress responses	Overexpression of mtDNA	[76]
Melasma	Impaired mitophagy and disrupted autophagic flux lead to the accumulation of damaged mitochondria	Increased ROS generation, resulting in oxidative stress and redox imbalance	Damaged mitochondrial renewal	[77]

*ROS* reactive oxygen species, *UV* Ultraviolet, *NF-κB* nuclear factor kappa-light-chain-enhancer of activated B cells, *TGF-β* transforming growth factor-β, *TCA* tricarboxylic acid

### Mitochondrion-targeted therapeutic strategies

Cutaneous mitochondrion-targeted therapeutic approaches involve diverse strategies designed to restore organelle homeostasis, although their clinical translation is hindered by challenges such as inefficient delivery systems, complex redox dynamics, and insufficient in vivo applicability.

#### Interventions to increase mitochondrial ATP production

Mitochondrial ATP synthesis in skin cells can be increased by activating key regulatory pathways and supplying essential metabolic cofactors. A central target is the transcriptional coactivator PGC-1α, which orchestrates mitochondrial biogenesis by engaging transcription factors such as NRF-1 and TFAM to promote the expression of mitochondrial proteins and mtDNA replication. Pharmacological activation of this axis, for example, through the use of rapamycin, has been shown to restore mitochondrial homeostasis and reduce cellular senescence in lung epithelial models, suggesting broader potential for ameliorating the age-related decrease in mitochondrial function [78].

Supplementation with metabolic precursors represents another route to increase the mitochondrial ATP output. L-Carnitine facilitates the transport of fatty acids into mitochondria for β-oxidation, thereby increasing substrate availability for oxidative phosphorylation. Coenzyme Q10 functions as a critical electron carrier in the electron transport chain and serves as a lipid-soluble antioxidant; coenzyme Q10 supplementation has been shown to improve mitochondrial bioenergetics and mitigate age-related cellular decline [79]. Furthermore, increasing the cellular levels of NAD^+^, a central coenzyme involved in redox reactions and energy metabolism, increases mitochondrial respiratory efficiency and ATP production, as evidenced by studies showing the restoration of cellular NAD^+^ levels in various tissues [80].

#### Antioxidants that reduce mitochondrial reactive oxygen species production

ROS, particularly those generated within mitochondria, play important roles in photoaging, inflammation, and a broad spectrum of skin pathologies. Antioxidants act to mitigate mitochondrial damage either by suppressing ROS formation or by neutralizing ROS once they are generated. Among antioxidants, mitochondrion-targeted antioxidants such as MitoQ and SkQ1 are engineered to accumulate within the organelle, increasing their ability to scavenge ROS and protect mitochondrial integrity. In HDFs exposed to ultraviolet A (UVA) or H_2_O_2_, MitoQ exerts protective effects on mtDNA damage, although a localized antioxidant such as tiron shows even greater efficacy under certain conditions [81]. SkQ1, another lipophilic cation-linked plastoquinone derivative, has similarly shown therapeutic potential; in animal models, it effectively normalizes inflammatory and regenerative wound-healing phases by preserving mitochondrial ultrastructure and reducing mtROS and mtDNA release [82]. In addition, compounds such as methylene blue have shown a potent ROS scavenging capacity in cultured human skin fibroblasts, including those derived from individuals with premature aging syndromes, reinforcing their potential for antiphotoaging applications [83].

While mitochondrion-targeted antioxidants offer targeted protection, nonspecific antioxidants such as vitamin C and vitamin E also contribute to protective effects by neutralizing ROS at the cellular level. These compounds indirectly support mitochondrial health by preserving redox homeostasis and limiting the propagation of oxidative damage in skin cells.

#### Bioactive compounds and drugs for mitochondrial protection and repair

Some bioactive compounds and drugs can maintain skin health by protecting mitochondria from damage or promoting mitochondrial DNA repair, and some bioactive compounds and drugs with the potential to protect and repair mitochondria are listed below.

(i) Mitochondrial protectors: These drugs protect mitochondria from damage by regulating cellular metabolism and autophagy pathways. For example, metformin promotes mitochondrial biogenesis and functional recovery by activating the AMPK pathway [84], and cyclosporin A can inhibit the opening of the mitochondrial permeability transition pore (mPTP), protect the integrity of the mitochondrial membrane, and prevent mitochondrial damage and apoptosis. Urolithin A and spermidine can maintain mitochondrial health and function by promoting autophagy and mitochondrial autophagy to remove damaged mitochondria [85].

(ii) Mitochondrial repair agents, such as NR and PQQ, can promote mitochondrial DNA repair and functional recovery by increasing NAD+ levels. Nicotinamide nucleoside is a precursor of NAD+ that can increase the level of NAD+ in cells, thereby enhancing mitochondrial function [86]. The experimental drug EPI-743 helps reduce oxidative stress and protect mitochondria by increasing the activity of glutathione [87], and positive results have been obtained with this drug in clinical trials for certain mitochondrial diseases.

Through these interventions, the function of mitochondria can be effectively improved, skin diseases caused by mitochondrial damage can be reduced, and the process of skin aging can be delayed [88].

#### Synergistic therapeutic strategies

While individual therapeutic approaches have shown promise in targeting mitochondrial dysfunction, combining multiple strategies may further increase efficacy, reduce the treatment duration, and lower the required dosages of individual agents. Synergistic therapies exploit the complementary mechanisms of action of different interventions. For example, the concurrent use of mitochondrion-targeted antioxidants (MitoQ) with mitochondrial biogenesis enhancers (NR or L-carnitine) can simultaneously reduce oxidative stress and increase energy production, providing a more comprehensive restoration of mitochondrial function [89, 90]. Similarly, combining autophagy activators (urolithin A) with agents that stabilize the mitochondrial membrane (cyclosporin A) may improve the clearance of damaged mitochondria while preserving the structural integrity of the mitochondrial network [91].

In certain skin disorders, integrating mitochondrion-targeted treatments with conventional dermatological therapies has potential benefits. For example, in psoriasis or AD, mitochondrion-targeted antioxidants used alongside biological immunomodulators may affect both the inflammatory cascade and the underlying mitochondrial oxidative stress [92, 93]. In pigmentary disorders, the coadministration of agents that improve mitochondrial respiration with topical treatments aimed at regulating melanin may increase pigment stabilization and reduce relapse rates [94]. Although clinical data on synergistic mitochondrial therapies in dermatology remain limited, preliminary studies and experimental models suggest that these combinations could offer greater therapeutic benefits than monotherapies.

#### Challenges and considerations for clinical translation

Despite the increasing interest in mitochondrion-targeted therapies for dermatological conditions, several significant challenges limit their clinical translation and long-term applicability. One major concern is the specificity and physiological relevance of antioxidants. While agents such as MitoQ or SkQ1 effectively scavenge mitochondrial ROS, the over suppression of ROS may interfere with essential redox signaling pathways required for normal cell function, wound healing, and immune modulation. Furthermore, the delivery efficiency of many antioxidants remains suboptimal, especially through topical administration where dermal penetration is limited, and mitochondrial accumulation may vary depending on the cell type and metabolic state [95, 96].

Mitochondrial protectants and metabolic modulators, including metformin, urolithin A, and NR, face hurdles related to tissue specificity, metabolic stability, and off-target effects. Their effects on mitochondrial function may differ across cell types or disease contexts, raising concerns about dosing and therapeutic windows that have yet to be thoroughly evaluated in human skin models. Similarly, mitochondrial transplantation (the transfer of healthy mitochondria into recipient cells) has performed favorably in animal models, such as models of diabetic wounds or ischemia–reperfusion injuries, but faces major translational barriers. These barriers include challenges in donor cell sourcing, immunogenicity, delivery systems, the integration of exogenous mitochondria, and long-term safety [97].

In the clinic, logistical gaps remain in terms of optimizing delivery routes (topical versus systemic), establishing effective dose regimens, and implementing patient stratification based on mitochondrial dysfunction phenotypes. The absence of reliable biomarkers to monitor the response to mitochondrion-targeted therapy further limits its application. Moreover, the translational gulf between preclinical animal models and human conditions cannot be overlooked because of species differences in mitochondrial density, skin architecture, metabolism, and immune responses. Economic and manufacturing considerations, including scalable production and cost-effectiveness for clinical use, also pose significant obstacles.

Addressing these challenges will require coordinated efforts to develop noninvasive mitochondrial biomarker platforms, advanced targeted delivery technologies, robust clinical trial designs with clearly defined end points, and regulatory-compliant manufacturing protocols. Only by closing these translational gaps can mitochondrion-targeted therapies move from exploratory research to practical treatments for skin diseases.

### Mitochondria and skin regeneration

Mitochondria are increasingly acknowledged as essential contributors to skin regeneration. In addition to their established function in energy generation, they participate in modulating inflammatory responses, stimulating angiogenesis, and orchestrating tissue remodeling. Accumulating evidence indicates a strong connection between mitochondrial metabolism, intercellular mitochondrial transfer, and the efficacy of wound healing. This section provides an in-depth examination of the functional involvement of mitochondria in skin repair and evaluates therapeutic strategies that leverage mitochondrial regulation to improve the regenerative potential of skin.

#### Role of mitochondria in skin wound healing

Mitochondria directly influence multiple stages of wound healing by regulating cellular metabolic states and energy production. Wang *et al.* reported that the transfer of exogenous mitochondria into adipose-derived mesenchymal stem cells (ADSCs) significantly increased their energy levels, thus improving the efficiency of cell engraftment [98]. Moreover, mitochondrial transfer markedly increases the bioenergetic metabolism of ADSCs, as evidenced by a significant increase in ATP levels, which provides the necessary energy support for effective wound healing [99].

During the wound healing process, mitochondrial metabolism in macrophages plays a key role in the inflammatory response in the early stage and the promotion of the repair process in the late stage. Macrophages in early-stage wounds produce ROS through mitochondrial activity, specifically mitochondrial ROS (mtROS), and drive the angiogenic program through the stabilization of HIF1a, a key factor required for timely healing [100]. In the later stages, macrophages shift toward a reliance on mitochondrial stress to promote regenerative responses [101].

Mitochondrial function is closely related to skin regeneration. Mitochondrial transfer has been shown to upregulate mRNAs associated with signaling pathways involved in DNA replication and cell division in ADSCs, thereby promoting the proliferation, migration, and differentiation of ADSCs in vitro [102]. In addition, alterations in mitochondrial metabolism are considered key mechanisms regulating the repair functions of macrophages at different stages of wound healing. For example, in macrophages present in early-stage wounds, the ETC enhances inflammatory responses and angiogenesis through the production of mtROS. In later stages, macrophages support tissue repair via IL-4RA-mediated mitochondrial respiration and mitohormesis [103].

#### Strategies to promote mitochondrial function to enhance skin regeneration

Regulating the mitochondrial metabolic pathway can increase the functional diversity of macrophages in wounds and may provide an effective strategy to promote the healthy repair of damaged tissues [104]. Furthermore, modulation of the mitochondrial stress response and maintenance of mitochondrial integrity may help extend the lifespan of tissue-resident macrophages, thereby enabling them to perform sustained tissue remodeling functions more effectively [105].

Another emerging strategy involves the use of engineered mitochondrial transfer to introduce healthy mitochondria into damaged cells, thereby increasing their energy production and repair capacity [106]. Yao *et al.* demonstrated that transferring mitochondria from young donors into ADSCs from elderly recipients significantly restored mitochondrial function and improved the skin repair capacity of the recipient cells [107].

Jiaxin Ding *et al.* investigated the incorporation of Au-EGCG into a hydrogel for the treatment of diabetic wounds. Animal experiments revealed that this strategy not only promoted the migration and proliferation of human umbilical vein endothelial cells but also reduced mitochondrial damage and oxidative stress in infected cells. The expression levels of bFGF and VEGF were significantly upregulated. After 12 days of treatment, the wound closure rate reached 97.2%, indicating a substantial acceleration in the healing of chronic diabetic wounds [108]. Optimizing culture conditions or applying growth factors and small-molecule compounds can further enhance mitochondrial function, thereby improving the regenerative capacity of skin cells.

### Multifaceted roles of mitochondria in skin biology and therapeutic horizons

Recent advances in mitochondrial biology have expanded our understanding of the functions of these organelles beyond their classical role in energy production, revealing broader regulatory functions in cutaneous physiology. Accumulating evidence underscores their essential involvement in cellular signaling cascades, redox homeostasis, and intercellular communication networks, highlighting their effects on a range of skin-related processes, such as epidermal aging, inflammatory responses, melanin synthesis, and wound repair (Table 2) [109–132].

**Table 2:** Skin disorders caused by mutations in nuclear DNA affecting mitochondrial function

Skin disease	Gene	Regulation	Mitochondrial pathology	Limitation	Adverse effects	Clinical applications	Ref.
Leukoderma	GABARAPL2 (GABA Type A Receptor-Associated Protein Like 2)	Upregulated	Involved in the later stage of autophagy maturation and induces autophagy	The functional role of GABARAPL2 in mitophagy and immunity is not yet fully defined; the current data are based mainly on in silico studies and qRT–PCR validation	No direct adverse effects are known, but nonspecific modulation may affect autophagy and IFN-γ pathways	High diagnostic value (AUC = 0.988); potential as a vitiligo biomarker, pending further validation	[109]
	SP1 (Sp1 Transcription Factor)	Downregulated	Interacts with E2F transcription factor 1 (E2F1), activates mitofusin2 (MFN2) expression, and regulates mitochondrial autophagy by regulating mitochondrial fusion	The role of SP1 has mainly been studied in vitro; its relevance in vivo and specificity remain uncertain due to its involvement in complex signaling cascades	Broad inhibition may interfere with essential transcriptional programs, causing unintended cellular effects	Potential target for modulating MFN2 expression and vascular remodeling; mithramycin A shows indirect therapeutic relevance	[110]
	USP8 (Ubiquitin-Specific Peptidase 8)	Downregulated	Ubiquitin conjugators lacking USP8 persist in Parkinson’s disease protein 2 (PARK2), delaying PARK2 translocation to damaged mitochondria and completion of mitochondrial autophagy	Studies focus on neurons; skin-specific roles remain unclear Dual effects on mitophagy and protein degradation complicate targeting	Inhibition may worsen α-synuclein accumulation and cytotoxicity	Potential target in neurodegenerative disease; not yet applied in skin disorders	[111]
	RELA (RELA Proto-Oncogene, NF-KB Subunit)	Upregulated	Affects NF-κB transcriptional mechanisms, regulates responses ranging from inflammation and immunity to cell growth, survival, and proliferation	Studied only in vitro; no in vivo or clinical data are available	No significant toxicity at 10 μM; higher doses of curcumin may be cytotoxic	NF-κB inhibition by curcumin/capsaicin may help prevent vitiligo progression	[112]
	TBC1D17 (TBC1 Domain Family Member 17P)	Downregulated	Deletion results in the loss of Rab7 localization to the damaged mitochondria, resulting in impaired mitochondrial autophagy	TBC1D17 was not directly studied; instead, TBC1D5 was identified as the critical GAP involved in regulating RAB7 during mitophagy. Thus, the function of TBC1D17 remains unclear in this context.	The loss of TBC1D5 leads to RAB7 hyperactivation and mislocalization, resulting in impaired ATG9A trafficking and defective mitophagosome formation. If TBC1D17 has overlapping functions, its deletion might similarly disrupt mitochondrial autophagy.	Understanding TBC1D5-mediated RAB7 regulation provides insights into mitophagy defects relevant to neurodegenerative diseases, such as Parkinson’s disease	[109]
Psoriasis	TNF (Tumor Necrosis Factor)	Upregulated	Inhibits PINK1-mediated mitochondrial autophagy, leads to changes in mitochondrial function and increases cytoplasmic mtDNA levels	The evidence is mainly derived from in vitro and mouse models; limited clinical validation	TNF inhibition may impair host immunity and increase the infection risk	Targeting TNF may restore mitophagy and improve mitochondrial function in individuals with inflammatory diseases	[113]

(*Continued*)

**Table 2:** Continued

Skin disease	Gene	Regulation	Mitochondrial pathology	Limitation	Adverse effects	Clinical applications	Ref.
	MELK (Maternal Embryonic Leucine Zipper Kinase)	Upregulated	Involved in cell cycle regulation, cell proliferation, cell differentiation, cell survival, and plays a key role in splicing complex assembly, gene expression, embryonic development, and hematopoiesis	Most data are derived from HCC cells and mice; no clinical trials are available	No specific toxicity reported; off-target effects remain unclear	Targeting MELK may overcome resistance to elesclomol in liver cancer	[114]
Ataxia telangiectasia	ISG15 (ISG15 Ubiquitin-Like Modifier)	Upregulated	Increases oxidative stress and the levels of unhealthy mitochondria, inhibits mitochondrial autophagy	Impairs mitochondrial quality control	Promotes mitochondrial dysfunction and cellular stress	Potential target for neurodegenerative diseases like ataxia telangiectasia and ALS where ISG15 expression is abnormally elevated	[115]
	ATM (ATM Serine/Threonine Kinase)	Downregulated	Regulates the function of organelles such as mitochondria and peroxisomes, angiogenesis and glucose metabolism	ATM is downregulated, impairing the regulation of mitochondria, peroxisomes, angiogenesis, and glucose metabolism	Results in neurodegeneration, oxidative stress, metabolic disorders, immunodeficiency, and increased cancer susceptibility	Currently limited; diagnosis and management rely on symptom control, immunoglobulin therapy, and supportive care	[116]
Wound healing disorder	ANT2 (Adenosine nucleotide translocase 2)	Downregulated	Promotes wound healing by regulating cell proliferation, energy homeostasis, and inflammation	The data are mainly derived from aged mouse models; human relevance needs validation	No direct toxicity reported; overactivation may affect energy balance	ANT2 activation promotes wound healing by restoring mitochondrial function in aging skin	[117]
	E2F3 (E2F Transcription Factor 3)	Downregulated	Causes re-epithelialization disorder; its depletion attenuates hypoxia-induced mitochondrial autophagy and increases intracellular reactive oxygen species levels	Limited understanding of how E2F3 depletion affects mitochondrial autophagy under hypoxic conditions	Its downregulation causes re-epithelialization disorder and increases intracellular ROS levels	Its downregulation causes re-epithelialization disorder and increases intracellular ROS levels	[118]
	Cox10 (Cytochrome C Oxidase Assembly Factor Heme A:Farnesyltransferase COX10)	Downregulated	Its deficiency leads to the accumulation of TCA cycle intermediates and decreased levels of glycolytic intermediates, impairs the angiogenic capacity	The findings are based on endothelial-specific knockout mice; validation in humans is lacking	Cox10 deficiency impairs angiogenesis and mitochondrial metabolism, potentially worsening tissue repair	Targeting mitochondrial function may enhance vascular repair in individuals with metabolic or ischemic diseases	[119]
	SIRT6 (Sirtuin 6)	Downregulated	Specific defects affect the temporal response of the wound healing process by inhibiting epithelial regeneration, angiogenesis, and collagen deposition	The context-specific roles of SIRT6 remain unclear and require further mechanistic studies	SIRT6 deficiency leads to impaired wound healing, inflammation, and metabolic dysfunction	SIRT6 activation may improve wound repair, metabolic health, and delay aging	[120]
Keloid	NFE2L2 (NFE2-Like BZIP Transcription Factor 2)	Downregulated	Regulates ferroptosis by directly influencing GPX4 synthesis and function and the peroxisome proliferator activated receptor γ (PPARγ) pathway	The findings are based on animal and in vitro models; clinical validation in humans is lacking	NFE2L2 downregulation increases ferroptosis and exacerbates mitochondrial dysfunction	Targeting NFE2L2 may protect against doxorubicin-induced cardiomyopathy	[121]

(*Continued*)

**Table 2:** Continued

Skin disease	Gene	Regulation	Mitochondrial pathology	Limitation	Adverse effects	Clinical applications	Ref.
	GPX4 (Glutathione Peroxidase 4)	Downregulated	Inactivates lipid peroxides through GSH and then inhibits cell ferroptosis	The dependence of GPX4 on glutathione and its vulnerability to inactivation by oxidative stress or inhibitors like RSL-3 limits its robustness as a defense against ferroptosis	GPX4 inhibition or deficiency leads to uncontrolled lipid peroxidation and nonapoptotic cell death, contributing to tissue damage in conditions such as myocardial infarction and neurodegeneration	GPX4 inhibition or deficiency leads to uncontrolled lipid peroxidation and nonapoptotic cell death, contributing to tissue damage in conditions such as myocardial infarction and neurodegeneration	[122]
	MFNs (Mitofusins)	Upregulated	Control mitochondrial fusion/fission, and its high expression promotes mitochondrial enlargement and increased numbers of mitochondria	Imbalanced mitochondrial fusion/fission in keloid fibroblasts leads to dysfunctional oxidative phosphorylation under normoxic conditions	These mitochondrial defects result in excessive ROS accumulation and metabolic shifts promoting persistent fibroblast proliferation	Targeting mitochondrial dynamics (e.g. MFN1/MFN2/FIS1 modulation) may represent a novel therapeutic strategy for managing keloid overgrowth	[123]
	MIR21 (MicroRNA-21)	Upregulated	Overexpression of miR21 inhibits caspase-8 activation and mitochondria-mediated apoptosis signaling pathways	The detailed mechanisms of FasL-mediated caspase-8 activation by miR-21 in keloid fibroblasts remain unclear and require further investigation.	The overexpression of miR-21 suppresses apoptosis in keloid fibroblasts, potentially contributing to keloid pathogenesis.	miR-21 may serve as a promising therapeutic target for inducing apoptosis in keloid fibroblasts through regulation of the FasL–caspase-8–mitochondrial pathway	[124]
Scleroderma	TFAM (Transcription Factor A, Mitochondrial)	Downregulated	Results in reduced mitochondrial numbers and the accumulation of damaged mitochondria with mtDNA release, mtDNA deletion mutant accumulation, impaired oxidative phosphorylation and metabolic changes	The causality between TFAM downregulation and fibrosis progression requires further validation in clinical human samples beyond experimental models	The causality between TFAM downregulation and fibrosis progression requires further validation in clinical human samples beyond experimental models	Targeting TFAM pathways may represent a therapeutic strategy to mitigate fibroblast activation and fibrosis in individuals with systemic sclerosis	[125]
	SIRT3 (Sirtuin 3)	Downregulated	Blocks myofibroblast differentiation and pulmonary fibrosis by preventing TGF-β1 signaling and inhibiting ROS production, mitochondrial DNA damage, and Tgf-β1-induced myofibroblast differentiation	The findings are primarily based on preclinical models, and thus the therapeutic potential of SIRT3 in humans remains unproven.	No significant adverse effects were reported in the study, but the long-term safety of SIRT3 overexpression has not been fully evaluated.	SIRT3 activation may serve as a novel therapeutic strategy to prevent or reduce pulmonary fibrosis by protecting mitochondrial DNA integrity.	[126]
	OGG1 (8-Oxoguanine DNA Glycosylase)	Downregulated	Results in increased mtROS production and mtDNA damage	Downregulation of OGG1 impairs the repair of oxidative mtDNA damage, contributing to mitochondrial dysfunction	Increased mtROS levels and mtDNA damage can promote inflammation and fibrosis progression	Targeting OGG1 activity could be a potential strategy to prevent oxidative damage in mitochondrion-related fibrotic diseases	[127]

(*Continued*)

**Table 2:** Continued

Skin disease	Gene	Regulation	Mitochondrial pathology	Limitation	Adverse effects	Clinical applications	Ref.
	Cox7C (Cytochrome C Oxidase Subunit 7C)	Upregulated	Stabilizes Complex IV and promotes more efficient energy use through the respiratory body super complex	The study is limited by a small sample size, single-center design, and the lack of direct measurement of electron transport chain activity	No direct adverse effects were reported, but altered ETC gene expression may signal a risk of more severe disease progression in systemic sclerosis patients	Differential ETC gene expression, including increased Cox7C expression, may serve as a potential biomarker for identifying ME/CFS in early systemic sclerosis and predicting disease severity	[128]
Melanoma	ACSL3 (Acyl-CoA Synthetase Long Chain Family Member 3)	Upregulated	Inhibits ferroptosis and promotes the distant metastasis of tumors	The dual role of ACSL3 in promoting tumor progression and conferring drug resistance complicates its targeting in therapy.	The overexpression of ACSL3 increases oleic acid uptake, inhibits ferroptosis, and promotes distant metastasis, especially in melanoma and triple-negative breast cancer.	ACSL3 serves as a potential prognostic biomarker and therapeutic target in cancers such as NSCLC, melanoma, prostate, and breast cancer, particularly in assessing statin sensitivity and androgen-driven tumor growth.	[129]
	BRAF (B-Raf Proto-Oncogene, Serine/Threonine Kinase)	Downregulated	Inhibits oxidative phosphorylation and drives glycolysis, avoids reactive oxygen species production during respiration, and prevents cellular aging	BRAF (V600E) mutation promotes resistance to therapy by remodeling metabolism through a shift from oxidative phosphorylation, contributing to oncogene-induced senescence escape.	Targeting BRAF can lead to metabolic compensation that limits drug efficacy and contributes to drug resistance.	Combining BRAF inhibitors with metabolic modulators like phenformin or PDK inhibitors (e.g. DCA) may increase therapeutic efficacy in melanoma.	[130]
	SOX2 (SRY-Box Transcription Factor 2)	Downregulated	Decreased SOX2 expression causes melanoma cells to shift to glycolysis, resulting in environmental acidification and increased tumors aggressiveness.	The study was conducted entirely in vitro, which limits its direct applicability to in vivo or clinical settings.	SOX2 overexpression may contribute to drug resistance and increased metastatic potential due to increased oxidative metabolism.	Targeting SOX2 could represent a therapeutic strategy to shift melanoma metabolism away from oxidative phosphorylation and reduce tumor aggressiveness.	[131]
	MCL1 (MCL1 Apoptosis Regulator, BCL2 Family Member)	Upregulated	Maintains mitochondrial integrity, exerts antiapoptotic effects	The findings are based primarily on engineered HCT116 cell lines, which may limit their generalizability to other cell types or in vivo contexts.	Overexpression or inhibition of BCL-2 family proteins, including MCL-1, could disrupt apoptosis regulation and lead to unintended cell survival or death.	Targeting MCL-1 alongside BCL-xL with BH3 mimetics or specific inhibitors represents a potential therapeutic strategy for inducing apoptosis in cancer cells.	[132]

#### Roles of mitochondrial dynamics in energy metabolism, cell death and signaling

In recent years, the roles of mitochondrial dynamics in skin health and disease have received increasing attention. Mitochondrial dynamics refers to continuous morphological changes, including the processes of fission and fusion, that are critical for maintaining intracellular homeostasis [133]. Recent studies have highlighted the involvement of mitochondrial dynamics in the regulation of energy metabolism, cell death, and cellular signaling pathways [134]. Mitochondrial fission increases cellular responsiveness to stress, whereas mitochondrial fusion facilitates the exchange of mitochondrial DNA and proteins, thereby preserving mitochondrial function and promoting overall cellular health [135].

#### Development and application of mitochondrion-targeting drugs

The development and application of mitochondrion-targeted therapeutics represent a cutting-edge area in biomedical research. Certain antioxidants can directly scavenge the ROS generated by mitochondria, thereby protecting cells from oxidative damage [136]. Through the use of various targeting moieties, including small molecules, peptides, liposomes, and nanoparticles, mitochondrion-targeted nanodelivery strategies facilitate the selective localization of drugs within mitochondria. These approaches can exert therapeutic effects by interfering with cellular energy metabolism, modulating intracellular ROS levels, targeting mitochondrial proteins, altering mitochondrial DNA, inducing mitophagy, or enabling combination therapies [137].

#### Mitochondria exert sympathetic functions in the skin microenvironment

The role of mitochondria in skin cells extends beyond energy production, as they are also involved in regulating the local skin microenvironment and mediating intercellular communication. Emerging research suggests that mitochondria may participate in cell-to-cell signaling in the skin through the generation of ultraweak photon emission (UPE), a noninvasive biomarker that provides new insights into skin health and disease monitoring [138]. Notably, alterations in the ECM can affect intracellular homeostasis, including mitochondrial function, through specific molecular signaling pathways [139].

#### Skin microbiome–mitochondria interactions

Emerging research indicates a dynamic reciprocal relationship between cutaneous microbial communities and mitochondrial activity mediated by bioactive compounds and cellular energy signaling pathways. Microbe-derived metabolites such as butyrate, propionate, and acetate—collectively termed short-chain fatty acids—serve as key regulators of cellular energy metabolism through dual mechanisms. These compounds activate specific G protein-coupled receptors (FFAR2/3) and function as histone deacetylase (HDAC) inhibitors, thereby upregulating the production of PGC-1ɑ, the primary coordinator of mitochondrial generation and energy production pathways. Recent in vivo studies have demonstrated that butyrate supplementation promotes mitochondrial differentiation programs in keratinocytes, reinforcing epidermal barrier integrity in murine AD models [140].

In turn, mitochondrial metabolites such as succinate play pivotal immunomodulatory roles. The accumulation of succinate, whether from metabolic dysregulation or mitochondrial stress, stabilizes HIF-1ɑ by inhibiting prolyl hydroxylase activity; the stabilization of this protein triggers transcriptional programs that increase the production of inflammatory cytokines such as IL-1β, thereby linking mitochondrial dysfunction to immune activation [141]. Succinate can act extracellularly through succinate receptor 1 (SUCNR1), further shaping immune cell function and the inflammatory milieu.

Within the context of inflammatory skin disease, dysbiosis characterized by the overgrowth of *Staphylococcus aureus* may exacerbate mitochondrial oxidative stress in keratinocytes via TLR2 activation, inducing both NADPH oxidase (NOX)-derived and mitochondrial ROS production. This oxidative burden can trigger mitochondrial DNA release, subsequently activating the cGAS–STING pathway and inducing type I interferon responses that amplify epidermal inflammation and barrier compromise.

#### Mitochondrial–epigenetic interactions in skin biology

Mitochondrial metabolism directly fuels epigenetic processes through the supply of essential metabolic intermediates that modulate chromatin-modifying enzymes. Acetyl-CoA, derived from mitochondrial pyruvate metabolism and fatty acid oxidation, is exported to the nucleus, where it serves as the bulky acetyl donor for histone acetyltransferases (HATs), thereby increasing histone acetylation levels and promoting the transcription of genes central to mitochondrial biogenesis, oxidative phosphorylation, and epidermal differentiation [142]. Mitochondrial nicotinamide adenine dinucleotide (NAD^+^) functions as a critical cofactor for sirtuin deacetylases, particularly SIRT1–3, enabling the removal of acetyl groups from histones and regulatory proteins. Through this process, NAD^+^-dependent sirtuins influence stress responses and aging in skin cells by controlling chromatin accessibility and gene expression [143].

The levels of the mitochondrial TCA cycle intermediate metabolites α-ketoglutarate, succinate, and fumarate critically regulate the activity of α-ketoglutarate-dependent DNA and histone demethylases such as TET enzymes and Jumonji C-domain histone demethylases. Perturbations in these metabolic intermediates, such as elevated succinate levels or reduced α-ketoglutarate levels, can inhibit demethylase activity, leading to aberrant gene silencing or activation, with implications for epidermal aging, regenerative capacity, and inflammatory gene expression. While direct evidence in the skin is still emerging, analogous mechanisms have been strongly characterized during aging and in other tissues [144].

The emergent epigenetic process of histone lactylation adds further nuance to mitochondrial–epigenetic crosstalk. In skin fibroblasts, elevated lactate levels, whether they are derived from glycolysis or extracellular sources (PLLA fillers), drive the lactylation of histone lysine residues such as H3K18la and H4K12la and even affect nonhistone proteins. This lactylation has been shown to modulate the expression of genes that regulate the fibrotic response, autophagy, and collagen synthesis in fibroblasts in hypertrophic scars [145, 146] and to increase collagen production in skin rejuvenation systems through KAT8-mediated lactylation of latent-transforming growth factor beta-binding protein 1 (LTBP1) at lysine 752 [147, 148].

Mitochondrial metabolites (acetyl-CoA, NAD^+^, TCA intermediates, and lactate) dynamically regulate epigenetic enzyme activity, histone marks, and the expression of genes relevant to skin homeostasis, aging, wound healing, and pathological remodeling. Such metabolite-driven chromatin changes and subsequent gene regulation suggest therapeutic approaches, including the manipulation of metabolic fluxes (NAD^+^ boosters and acetyl-CoA precursors), sirtuin activators, or lactylation modulators, with the potential to modulate skin aging, inflammation, and fibrosis.

The current evidence has established a close link between mitochondrial dysfunction, such as mtDNA mutations, ROS overproduction, and impaired mitophagy, and skin barrier disruption, chronic inflammation, pigment alterations, and tumorigenesis. These findings have collectively expanded the theoretical framework for understanding the roles of mitochondria in skin physiology and disease (Table 3) [149–157].

**Table 3:** Targeted treatment of dermatosis

Skin disease	Targeted drug	Therapeutic mechanism	Effect	Ref.
Leukoderma	Capsaicin	Combination treatment with MSCs inhibits TLR4 through HSP70, and the mTOR/FAK signaling pathway is inhibited	Alleviates mitochondrial dysfunction and abnormal autophagy in PIG3V cells	[149]
Psoriasis	TCeO2	Targets mitochondria and removes ROS	Reduces inflammation levels in cells and alleviates oxidative stress	[150]
	*Coleus vettiveroides* (CV)	Changes the distribution of actin microfilaments in HepG2 cells and leads to cell cycle stagnation in sub G0/G1 stage	Induces mitochondrial dysfunction, oxidative stress, cytoskeletal disorganization, cell cycle arrest, and mitochondria-mediated apoptosis	[151]
Ataxia telangiectasia	Nicotinamide riboside (NR)	Increases intracellular NAD levels	Promotes mitochondrial autophagy	[152]
	Enanthate	Enhances ER–mitochondrial contact and increases calcium flow from the ER to mitochondria, restores normal mitochondrial function and mitochondrial autophagy	Increase the resistance of ATM-deficient cells and cells from patients with A-T to metabolic stress-induced killing	[153]
Wound healing disorder	δ-TT-BMSCs	Reduces lipid ROS levels in NIH-3 T3 and PAM-212 cells and increases the mitochondrial membrane potential, promoting wound healing by reducing ferroptosis	Promotes cell proliferation, migration and angiogenesis	[154]
	Mesenchymal stem cell-derived extracellular vesicles (MSC-EVs)	Transfer functional mitochondria to neutrophils in wound tissue, inhibit NET formation and EC ferroptosis, and activate the PI3K/AKT pathway	Prevent impaired angiogenesis in diabetic wounds	[155]
Keloid	ADSC exosomes	Inhibit the PI3K/AKT/mTOR signaling pathway and promote mitochondrial autophagy	Improve mitochondrial morphology, reduce inflammation and cellular fibrosis	[156]
Melanoma	ZWZ-3	Upregulates LC-3II and Atg5 and downregulates P62 to induce autophagy	Induces apoptosis and inhibits the proliferation of tumor cells	[157]

## Conclusions

Over the past decade, mitochondrial biology has attracted substantial attention in dermatology research. In addition to their traditional role in ATP production, mitochondria are now recognized as regulators of skin cell differentiation, apoptosis, redox balance, and innate immune responses. However, significant limitations exist in the current body of research that constrain both the mechanistic depth and translational progress. Several research directions should be considered to address these challenges. First, the development of mitochondria-targeting tools with increased precision, including stimuli-responsive systems (pH, ROS, or enzyme-activated) and gene-based vectors with cell-type selectivity, is urgently needed. Second, multiomics approaches, such as mitochondrial genomics, metabolomics, and epigenomics combined with single-cell transcriptomic profiling, should be employed to characterize disease-specific mitochondrial signatures and reveal cell type-specific vulnerabilities. Third, efforts should be directed toward the identification and validation of noninvasive or circulating mitochondrial biomarkers. Fourth, advanced delivery strategies, like lipid nanoparticles, should be optimized to improve the targeting efficiency and stability of mitochondrial agents in human skin. Fifth, further investigations into the microbiome–mitochondria axis may reveal novel links between microbial metabolites (SCFAs and LPS) and mitochondrial signaling. Finally, rigorous preclinical models and early-phase clinical trials should be established to evaluate the safety, pharmacodynamics, and potential for combination regimens of mitochondrion-targeted therapies. In conclusion, while significant progress has been made in revealing the role of mitochondria in skin biology, the field now requires a shift toward more refined experimental models, mechanistic specificity, and translational viability. Addressing these needs will be key to establishing the mitochondria as credible and effective targets in future dermatological therapy.
